# Further characterization of *Shigella*-specific (memory) B cells induced in healthy volunteer recipients of SF2a-TT15, a *Shigella flexneri* 2a synthetic glycan-based vaccine candidate

**DOI:** 10.3389/fimmu.2023.1291664

**Published:** 2023-10-31

**Authors:** Franklin R. Toapanta, Jingping Hu, Shiri Meron-Sudai, Laurence A. Mulard, Armelle Phalipon, Dani Cohen, Marcelo B. Sztein

**Affiliations:** ^1^ Department of Medicine and Center for Vaccine Development and Global Health, University of Maryland School of Medicine, Baltimore, MD, United States; ^2^ School of Public Health, Faculty of Medicine, Tel Aviv University, Tel Aviv, Israel; ^3^ Institut Pasteur, Université Paris Cité, CNRS UMR3523, Unité Chimie des Biomolécules, Paris, France; ^4^ Institut Pasteur, Université Paris Cité, Laboratoire Innovation: Vaccins, Paris, France; ^5^ Department of Pediatrics and Center for Vaccine Development and Global Health, University of Maryland School of Medicine, Baltimore, MD, United States

**Keywords:** *Shigella*, conjugate vaccine, synthetic carbohydrate, memory B cells, antigen-specific B cells

## Abstract

Shigellosis is common worldwide, and it causes significant morbidity and mortality mainly in young children in low- and middle- income countries. To date, there are not broadly available licensed *Shigella* vaccines. A novel type of conjugate vaccine candidate, SF2a-TT15, was developed against *S. flexneri* serotype 2a (SF2a). SF2a-TT15 is composed of a synthetic 15mer oligosaccharide, designed to act as a functional mimic of the SF2a O-antigen and covalently linked to tetanus toxoid (TT). SF2a-TT15 was recently shown to be safe and immunogenic in a Phase 1 clinical trial, inducing specific memory B cells and sustained antibody response up to three years after the last injection. In this manuscript, we advance the study of B cell responses to parenteral administration of SF2a-TT15 to identify SF2a LPS-specific B cells (SF2a+ B cells) using fluorescently labeled bacteria. SF2a+ B cells were identified mainly within class-switched B cells (SwB cells) in volunteers vaccinated with SF2a-TT15 adjuvanted or not with aluminium hydroxide (alum), but not in placebo recipients. These cells expressed high levels of CXCR3 and low levels of CD21 suggesting an activated phenotype likely to represent the recently described effector memory B cells. IgG SF2a+ SwB cells were more abundant than IgA SF2a + SwB cells. SF2a+ B cells were also identified in polyclonally stimulated B cells (antibody secreting cells (ASC)-transformed). SF2a+ ASC-SwB cells largely maintained the activated phenotype (CXCR3 high, CD21 low). They expressed high levels of CD71 and integrin α4β7, suggesting a high proliferation rate and ability to migrate to gut associated lymphoid tissues. Finally, ELISpot analysis showed that ASC produced anti-SF2a LPS IgG and IgA antibodies. In summary, this methodology confirms the ability of SF2a-TT15 to induce long-lived memory B cells, initially identified by ELISpots, which remain identifiable in blood up to 140 days following vaccination. Our findings expand and complement the memory B cell data previously reported in the Phase 1 trial and provide detailed information on the immunophenotypic characteristics of these cells. Moreover, this methodology opens the door to future studies at the single-cell level to better characterize the development of B cell immunity to *Shigella*.

## Introduction

Shigellosis continues to be a significant public health problem, especially in low- and middle-income countries (LMICs) where children under 5 years of age are particularly susceptible. Over 200 million cases of shigellosis occur every year in LMIC, with ~200,000 deaths reported and ~64,000 of these among young children (<5 years-old) (1). Even when not deadly, repeated bouts of diarrheal disease impair physical growth and cognitive abilities (2, 3). *S. flexneri* is the leading cause of endemic shigellosis in LMICs (~60% of infections) (4), while *S. sonnei* is the second most common cause (~25% of episodes). *S. sonnei* is also the leading species in high-income countries (5) and outbreaks are linked to i) young children in child-care facilities/schools (6), ii) travelers and military personnel (7, 8) and iii) men who have sex with men (9–11). Outbreaks caused by multidrug-resistant *Shigella* have also been reported in several high-income countries (12, 13). The main prevention strategy for shigellosis includes improved sanitation, access to clean water sources and good personal hygiene. However, *Shigella* requires a low bacteria inoculum to cause disease (100-1000 bacilli) which leads to frequent failures of preventive sanitary measures. Additionally, *Shigella* is classified as a category B agent of biodefense concern, making it a priority for the development of therapeutics and vaccines (14).

It is possible to induce protective immunity against shigellosis. For example, naturally-acquired wild-type (wt) *Shigella* infection confers ~70% serotype-specific immunity (15–17). Moreover, adults experimentally infected with either *S. sonnei* or *S. flexneri* are significantly protected against illness following re-challenge with the homologous strain (64-74% efficacy) (18, 19). Anti-lipopolysaccharide (LPS) antibodies appear to be critical for protection (17, 20). Despite great efforts, no licensed vaccine for shigellosis is available. Most vaccine efforts have focused on the O-antigen, a polysaccharide unit of the outer membrane LPS (21–24). *Shigella* detoxified LPS-protein conjugates have shown to be a potentially viable strategy (22, 25, 26). As a lead demonstration, a *S. sonnei* conjugate showed 74% protective efficacy in young adults (26) and 71% protective efficacy in children aged 3-4 years but was not protective in younger children (27), highlighting the need to develop improved vaccines. A novel sub-type of conjugate vaccine candidate was recently proposed. This vaccine is composed of a synthetic 15mer oligosaccharide (OS), designed to act as a functional mimic of the *S. flexneri* serotype 2a (SF2a) O-antigen and covalently linked to tetanus toxoid (TT) via single point attachment (herein SF2a-TT15) (24, 28). This synthetic carbohydrate-based vaccine candidate was shown to be safe and immunogenic following parenteral administration in a recent Phase I clinical trial (21). Given the importance of humoral responses in protection (29), especially antibodies against the O-antigen, it is critical to understand better how these responses are developed and persist over time, as well as the characteristics of the cells responsible for producing class-switched antibodies. In this manuscript, we expand our understanding of the B memory responses elicited by SF2a-TT15 by using a new method for the identification of *Shigella-*LPS-specific B cells.

## Materials and methods

### Labeling of whole bacteria with fluorescent dyes

SF2a (2457T) from a CVD master stock was grown into LB agar (overnight; 37°C). Next morning, cells were gently harvested from the plate using a cell scraper and 5 mL of 1X PBS. Bacteria were transferred to a 50 ml conical tube. The harvesting procedure was repeated twice and 1X PBS was added to the tube for a total of 20 mL. Bacteria was then placed into a water bath (65°C) for 60 minutes and then transferred to iced water for 5 minutes. Two mL of 10% formaldehyde were added to the bacteria and incubated at room temperature for 1 hour. The bacteria concentration was adjusted to 0.4 (O.D. 600 nm) using 1X PBS. Ten mL of bacteria (0.4 at 600 nm) were transferred to a 15 mL conical tube and centrifuged (4,300 g, 30 min at 4°C). The supernatant was carefully removed, the bacteria pellet resuspended in 15 mL of 1X PBS and centrifuged again (4,300 g, 30 minutes at 4 °C). The supernatant was removed, and the bacteria cell pellet resuspended in 0.5 mL of Pacific Blue succinimidyl ester (Molecular Probes, Eugene, OR) at a concentration of 20 μg/mL in 1X PBS. Bacteria were stained for 1 hour at room temperature (RT) in the dark. Then, bacteria were washed with 10 mL of a solution of 1% FBS in 1X PBS (4,300 g, 30 minutes at 4°C). The supernatant was discarded, the bacteria pellet was resuspended in a solution of 1% formaldehyde in 1X PBS and incubated for 1 hour at RT. The bacteria pellet was then washed twice with 10 mL of 1X PBS (4,300 g, 30 minutes at 4°C). The bacteria pellet was then resuspended in 3 mL of 1% BSA/1XPBS and the O.D. (600 nm) adjusted to 0.4. SF2a-Pacific Blue (SF2a-PB) was stored at 4°C covered from light. A similar procedure was used to label a second set of SF2a with Alexa Fluor 488 succinimidyl ester (Molecular Probes, Eugene, OR). Successful labeling of the bacteria was tested in an LSR-II flow cytometer (Beckton Dickinson, NJ) (**Supplementary Figures 1A-C**) using unlabeled bacteria as control. Only bacteria preparations with >90% labeled bacteria were considered appropriate for the experiments presented in this manuscript.

### Ethics statements and clinical samples

The specimens used in this study were collected as part of a Phase I clinical trial assessing the safety and immunogenicity of SF2a-TT15, a synthetic carbohydrate conjugate vaccine against SF2a (28). The clinical component of the study was performed at the Clinical Research Center of Tel Aviv Sourasky Medical Center (Israel). All volunteers provided informed consent. The study is registered with ClinicalStudies.gov, NCT02797236 and has been completed. Peripheral blood mononuclear cells (PBMC) were isolated from vaccine and placebo recipients at days 0, 28, 56, 84 and 140 (**Supplementary Figure 1D**) using a standard Ficoll density fractionation method and cryopreserved. De-identified specimens (PBMC) from six volunteers who received SF2a-TT15 (10 µg), six that received SF2a-TT15 + Alum (10 µg) and four that received Placebo + Alum were assessed at the University of Maryland School of Medicine. Safety and immunogenicity data (humoral response) have already been reported (21).

### Flow cytometry staining for SF2a-specific B cells

**Figure 1:**
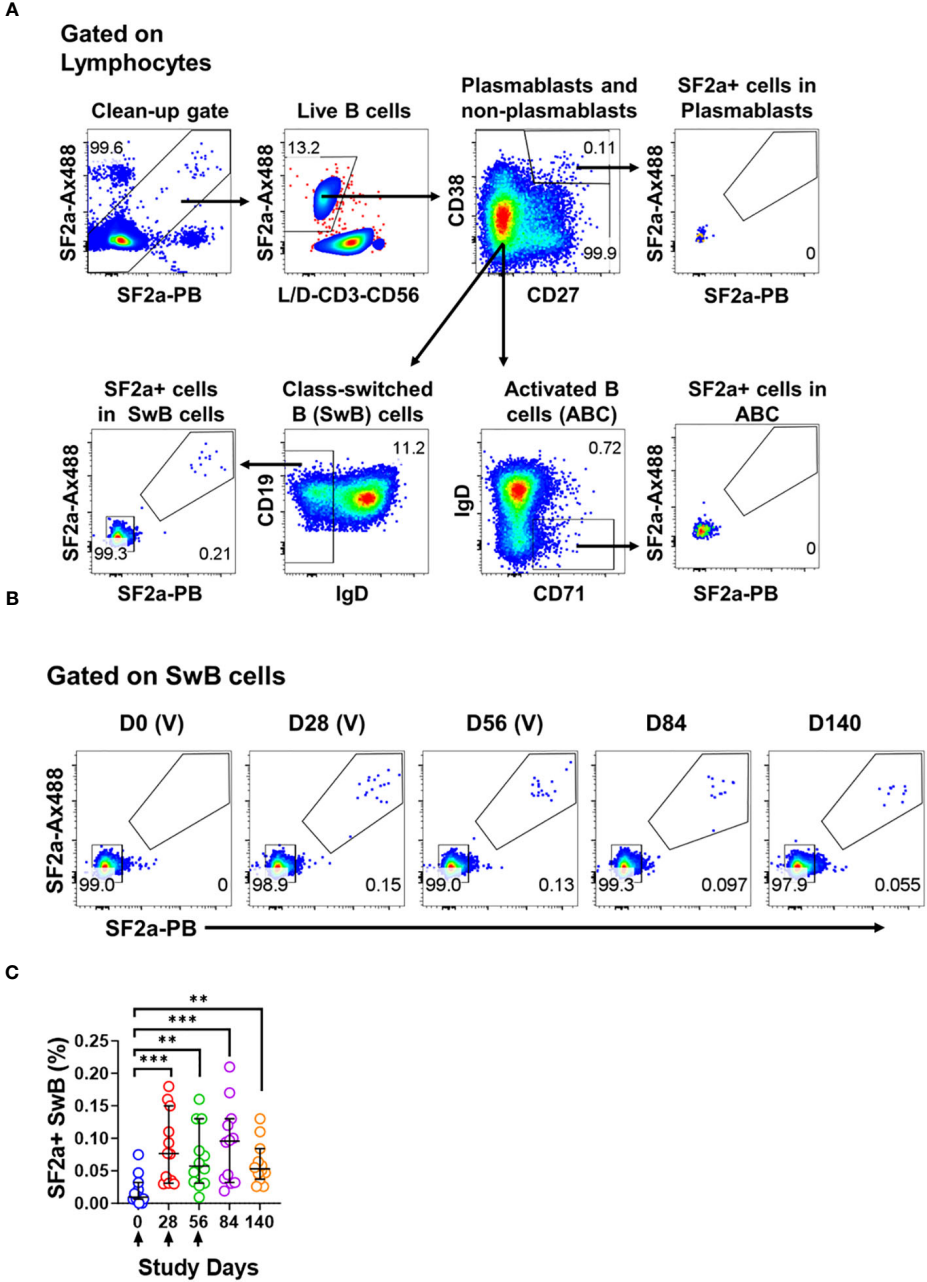
Identification of SF2a+ B cells in PBMC by flow cytometry. Panel **(A)** shows the gating strategy used to identify various B cell subsets including Plasmablasts (CD3- CD19+ CD38++, CD27++), Activated B cells (ABC; CD3- CD19+ CD27+/dim, CD38+/dim, IgD-, CD71+) and Switched B (SwB; CD3- CD19+ CD27+/dim, CD38+/dim, IgD-) cells. Note that this gating strategy eliminates non-specific SF2a binders using a clean-up gate (Top panel on the left and **Supplementary Figures 1D, E**). Panel **(B)** shows the identification and persistence of SF2a+ cells in SwB cells after vaccination in PBMC of a representative volunteer. Vaccine days are indicated as (V). Panel **(C)** shows the percentage of SF2a+ cells within SwB cells in volunteers that received SF2a-TT15 +/- Alum (n=12). Arrows show the vaccination days. Mean and 95CI are shown. The p values were calculated using unpaired t-tests (2-sided). **p<0.005, ***p<0.001.

**Figure 2:**
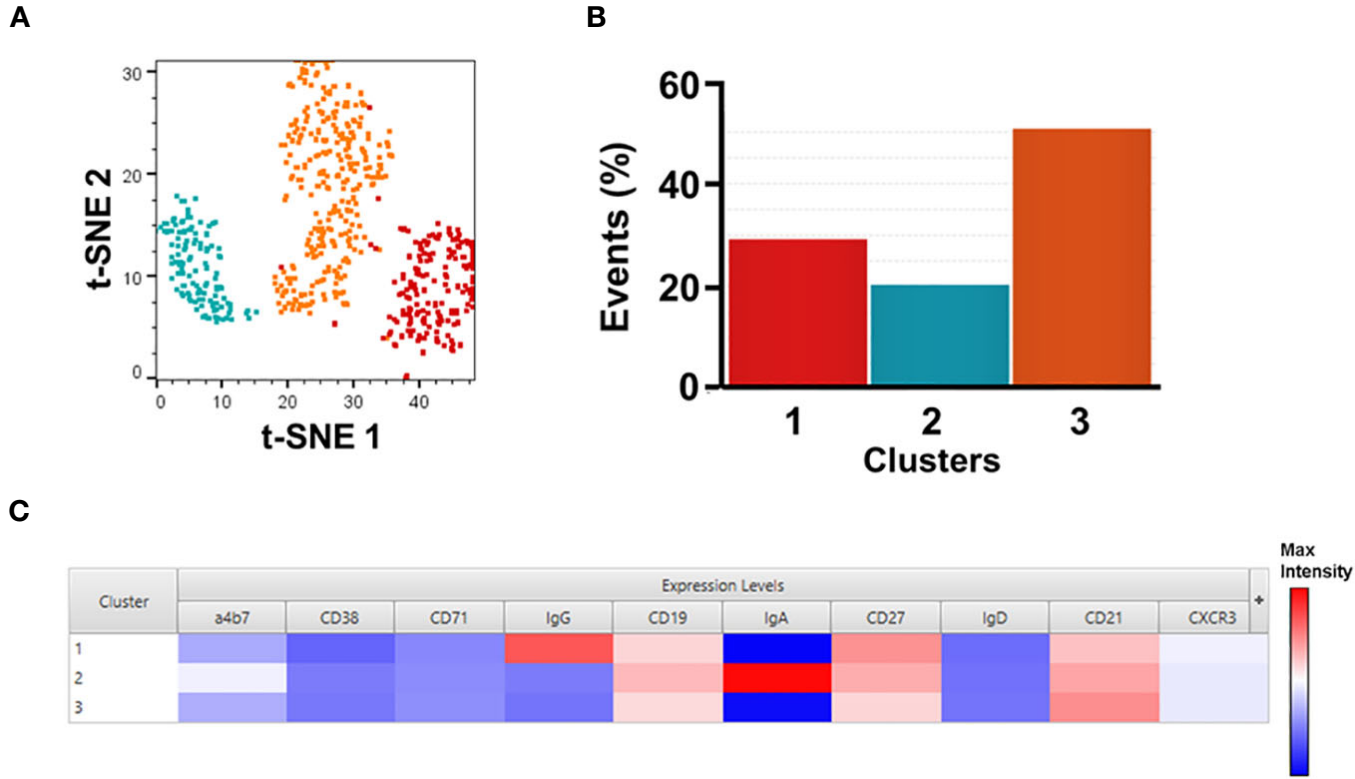
Unsupervised analysis of SF2a+ Sw B cells. Panel **(A)** shows t-SNE analysis overlaid with the X-shift analysis. The SF2a+ SwB cell population (concatenated) from all SF2a-TT15 +/- Alum vaccinated volunteers days 28-140 (no day 0) were used in the analysis. Panel **(B)** shows the frequency of the three cell clusters identified in the t-SNE/X-shift analysis. Panel **(C)** shows the expression intensity of the markers assessed (Cluster Explorer) and used to define the clusters.

Cryopreserved PBMC were thawed in complete RPMI (cRPMI) [RPMI (Gibco, NY, USA) supplemented with 10% fetal bovine serum (FBS) (Gemini Bioproducts, West Sacramento, CA), 2 mM L-glutamine (Gibco, Grand Island, NY, USA), 1x non-essential amino acids (Gibco, Grand Island, NY, USA), 10 mM HEPES (Gibco, Grand Island, NY, USA), 2.5 mM sodium pyruvate, (Lonza, Walkersville, MD, USA), 100 U/mL penicillin, 100 ug/ml streptomycin (Sigma-Aldrich, St. Louis, MO, USA), 50 μg/mL gentamicin (Gibco, Grand Island, NY, USA)]. Cells were immediately stained for flow-cytometry using methods previously described (30–34). Briefly, 2x10e6 cells were plated into 12x75 mm tubes, washed twice with 1X PBS (450 g, 5 min, 4°C) and stained for viability (20 min on ice) using 100 μL of a fixable yellow staining dye (Invitrogen, USA). After 2 washes with flow cytometry staining buffer (FCSB) (0.5% FCS in 1X PBS), cells were blocked with human Fc Block (BD Pharmingen, USA). Cells were then washed with FCSB (450 g, 5 min, 4°C) and resuspended in 50 μL of a solution containing 0.6 μL of SF2a-Ax488 (O.D. 0.4 at 600 nm) plus 0.6 μL of SF2a-PB (O.D. 0.4 at 600 nm) in FCSB. PBMC were stained with bacteria for 30 minutes at RT. After 2 washes with FCSB, the cells were stained with an antibody cocktail prepared in FCSB (30 min, RT). Monoclonal antibodies (mAbs) against the following molecules were used: IgA-PE (goal polyclonal; SouthernBiotech), CD19-ECD (clone J3-119; Beckman Coulter -BC-), IgD-PerCP-Cy5.5 (clone IA6-2; BD), CD27-PE-Cy7 (clone M-T271; BD), CD14-Brillant Violet 570 (BV570) (clone M5E2; BioLegend), CD3-BV570 (clone UCHT1; BioLegend), CD56-BV570 (clone HCD56; BioLegend), IgG-BV605 (clone G18-154; BD), CD21-BV711 (clone B-ly4; BD), CXCR3-BV785 (clone 1C6/CXCR3; BD), integrin α4β7-Alexa647 (clone ACT-1; Millennium, The Takeda Oncology Co), CD38-APC-R700 (clone LS1298-4-3; BD), CD71-APC-H7 (clone M-A712; BD). After washing with FCSB, the cells were fixed with 1% PFA in 1X PBS. Samples were collected in a custom LSRII flow cytometer (BD, USA). Samples were analyzed using FlowJo (Tree Star, San Francisco, CA) (**Figures 1A**, **2A**).

### Differentiation of B cells into antibody secreting cells and staining for SF2a-specific B cells

**Figure 3:**
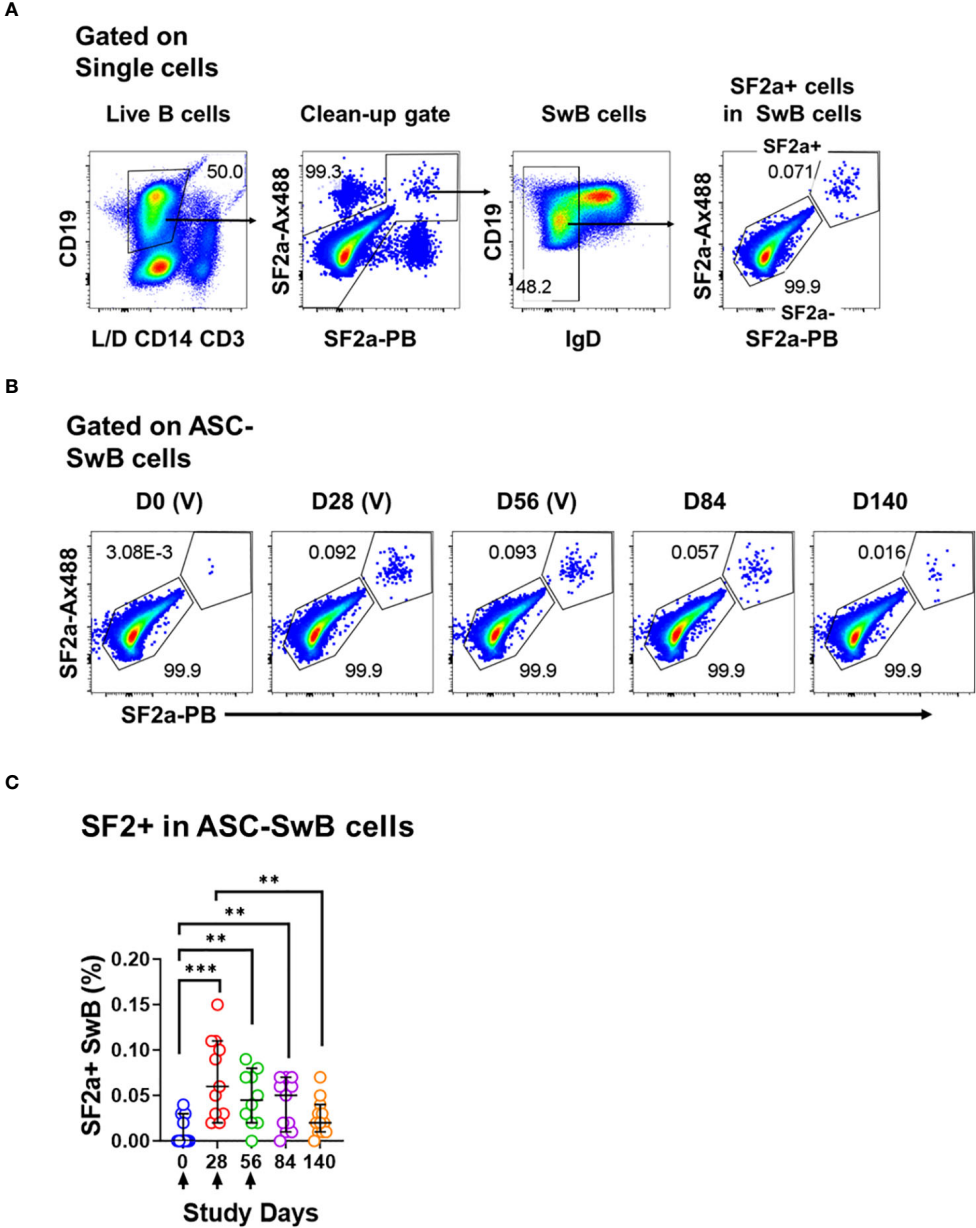
Identification of SF2a+ B cells in ASC by flow cytometry. **(A)** Shows the gating strategy used to identify SF2+ cells within SwB cells in polyclonally expanded B cells (ASC-transformed). Note that the gating strategy is different than the one used in PBMC. **(B)** Shows the identification and persistence of SF2a+ cells within SwB cells in ASC-transformed cells in a SF2a-TT15 +/- Alum representative volunteer. **(C)** Shows the percentage of SF2a+ cells within SwB cells in volunteers that received SF2a-TT15 +/- Alum (n=12). Arrows in X axis show the vaccination days. Median and 95CI are shown. The p values were calculated using unpaired t-tests (2-sided). **p<0.005, ***p<0.001, ****p<0.0001.

To differentiate B cells into ASC, PBMC were polyclonally expanded as described by Crotty et al. (35). In short, cryopreserved PBMC were thawed, washed with cRPMI and expanded for 5-6 days in 6-well sterile plates (2.5x10e6 cells/well) in the presence of 50 μM 2-mercaptoethanol, 1/100,000 pokeweed mitogen (PWM) (kindly provided by Dr. S. Crotty), 6 μg/mL CpG-2006 (Invivogen, CA), and 1/10,000 *Staphylococcus aureus* Cowan (SAC) (Sigma-Aldrich, St. Louis, MO) in cRPMI in a total volume of 2.5 mL/well. Cells were fed by adding an additional 2 mL of cRPMI after 2-3 days of incubation. On day 5-6 polyclonally stimulated cells were harvested, 2x10e6 cells plated into 12x75 mm tubes and stained for flow cytometry as described above. A similar panel to the one previously described was used. Samples were collected in a custom LSRII flow cytometer (BD, USA). Samples were analyzed using FlowJo (Tree Star, San Francisco, CA) (**Figure 3A**).

### B memory assay

Antibody production by activated and ASC differentiated B memory cells were measured using an optimized ELISpot assay for the measurement of IgA spot forming cells (SFC). In short, after 5-6 days of polyclonal stimulation, PBMC (see above) were harvested, counted and seeded in duplicate wells of multi-screen (Cellulose Esters membrane) plates (Millipore, USA) coated with SF2a LPS (Institut Pasteur, France) or goat-anti-human IgA and IgG (Total IgA and IgG control) (Jackson ImmunoResearch, PA) as previously described (35–38). After 5 hours incubation (37°C, 5% CO_2_), plates were incubated with goat-anti-human IgA- and IgG- horseradish peroxidase (Jackson ImmunoResearch, PA). IgA and IgG SFC were visualized with 3-amino-9-ethylcarbazole (AEC) substrate (Calbiochem, MA). SFC were enumerated using an automated ELISpot reader (Immunospot 3B, Cellular Technologies Ltd, OH) with aid of Immunospot software version 5.0 (Cellular Technologies Ltd). Total and antigen-specific B memory SFC were calculated as SFC/10e6 cells.

### Statistical methods

The D’Agostino and Pearson test was used to determine whether the data was normally distributed. The hypotheses in the study were evaluated using two sided tests and p values <0.05 without adjustment for multiple comparisons were considered statistically significant. The frequency of the cells or percentages of the various B cell subsets in vaccinated and placebo volunteers with normally distributed data were compared using unpaired student’s t-tests. Data sets without normal distribution (skewed) comparisons were done using Mann-Whitney tests. Statistical tests were performed in GraphPad Prism v9.3.0 (Boston, MA).

## Results

### *Shigella*-specific B cells in circulation after vaccination with SF2a-TT15

We used a method previously developed in our lab to efficiently label killed bacteria with fluorescent dyes (34). For all experiments described in this manuscript, we used *S. flexneri* 2a (2457T), a wild-type strain used in human challenges (19, 39, 40), labeled with Alexa Fluor 488 (SF2a-Ax488) or Pacific Blue (SF2a-PB) (**Supplementary Figure 1C**). These bacteria were used as “bait” to identify *Shigella*-binding B cells (henceforth SF2a-specific B cells or SF2a+ B cells) in specimens from Israeli volunteers who participated in a Phase 1 clinical trial that assessed the conjugate vaccine candidate SF2a-TT15 (21). Volunteers received three injections of SF2a-TT15 adjuvanted or not with Alum on days 0, 28 and 56. Peripheral blood mononuclear cells (PBMC) were collected on days 0, 28, 56, 84 and 140 (**Supplementary Figure 1D**). Safety and immunogenicity (humoral immunity) were reported elsewhere (21). In the current study, we assessed PBMC from volunteers who had received SF2a-TT15 +/- Alum (10 µg OS dose) (n=12) or the adjuvanted Placebo (Placebo + Alum, n=4).

To assess *Shigella*-specific B cells, we used equal amounts of bacteria labeled with each fluorochrome (Ax488 and PB; dual tag system). The bacteria were used in conjunction with a high-color flow cytometry panel to characterize different memory B cell subsets including plasmablasts (CD3- CD19+ CD27++ CD38++), activated B cells (ABC; CD3- CD19+ CD27dim/+ CD38dim/+ IgD- CD71+) and class-switched B cells (SwB; CD3- CD19- CD27dim/+ CD38dim/+ IgD-) (**Figure 1A**). In the gating strategy, we included a gate to eliminate non-specific bacteria binders (**Figure 1A**; **Supplementary Figures 1E, F**). The use of antigens labeled with two different fluorochromes has been proven useful to remove confounding factors such as B cells binding to the fluorochromes (41, 42).

SF2a+ cells were identified within SwB cells (memory B cells) (**Figures 1A, B**). Cohen et al, reported that the groups vaccinated with the 10µg dose of SF2a-TT15, adjuvanted or not, developed comparable anti-LPS IgG antibody titers (21). Similarly, the frequency of SF2a+ cells within SwB cells after day 0 was comparable in these two groups. Hence, in this manuscript, we report the data from these two groups assembled into one group (SF2a-TT15 +/- Alum; n=12). The compiled data showed that SF2a + SwB cells increased significantly after the first immunization (**Figures 1B, C**) and remained elevated until day 140. The frequency of these cells was significantly higher compared to placebo volunteers on days 28-140 (**Supplementary Figure 2A**). Like the anti-LPS IgG antibody titers described by Cohen et al. in the Phase 1 trial (21), which did not increase after the vaccine boosters (days 28 and 56), we did not see an increase the frequency of Sf2a+ SwB cells. Small fluctuations in the frequency of these cells between days 28-140 were noted, but not statistical differences were identified (**Supplementary Table 1**).

### Immunophenotypic characteristics of *Shigella*-specific SwB cells

**Figure 4:**
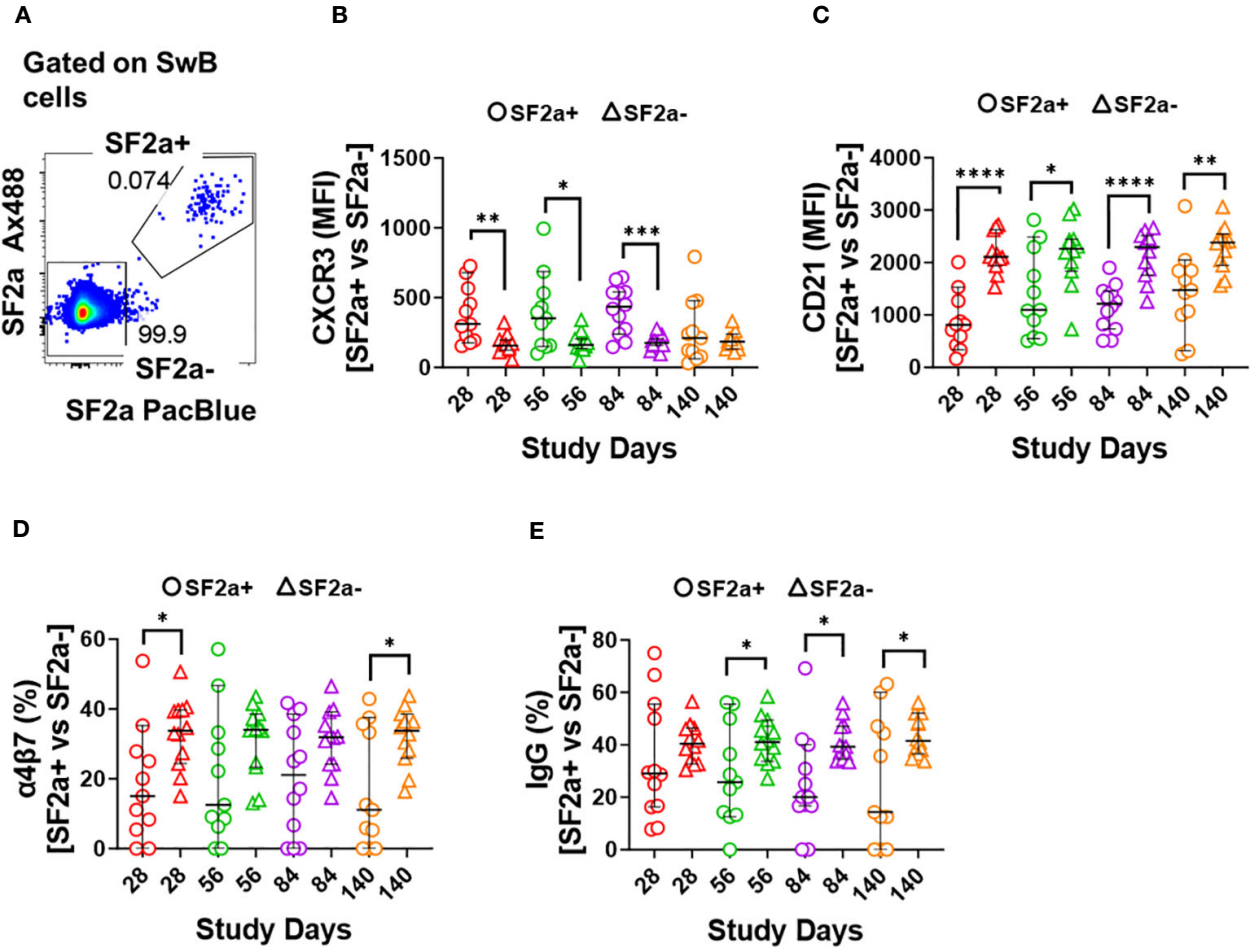
Characteristics of SF2a-specific B cells (SF2a+ SwB cells). Panel **(A)** shows an example of the gating used to identify SF2a+ and SF2a- cells within SwB cells. Data is from a representative volunteer first vaccine dose. Panels **(B, C)** show the Median Fluorescence Intensity (MFI) of CXCR3 and CD21, respectively, in SF2a+ and SF2a- SwB cells in vaccinated volunteers. Panels **(D, E)** display the percentage of SF2a+ and SF2a- SwB cells expressing integrin α4β7 and IgG, respectively. Panels **(B-E)** show the Median and 95CI. The p values were calculated using unpaired t-tests (2-sided). *p<0.05, **p<0.005, ***p<0.001, ****p<0.0001.

We further explored the characteristics of SF2a+ SwB cells by assessing markers associated with activation of antigen-specific B cells. CXCR3 is a chemokine receptor whose expression endows cells with the potential to home to inflamed tissues (43–46) and denotes activation. Downregulation of CD21 has been associated with cell activation and transitioning to the ASC stage (47, 48). We compared expression of CXCR3 and CD21 between SF2a+ and SF2a- (bacteria binders and non-binders, respectively) SwB cells in vaccinated volunteers (**Figures 4A-C**). Of note, on day 0, SF2a+ SwB cells were virtually absent therefore, comparisons of these markers with SF2a- SwB on this day were not possible. SF2a+ SwB cells showed higher expression levels of CXCR3 than SF2a- SwB cells on days 28, 56 and 84 (**Figure 4B**). In contrast, CD21 was downregulated in SF2a+ SwB cells on days 28, 56, 84 and 140 (**Figure 4C**). No differences in the expression of CD21 and CXCR3 between days 28-140 in SF2a+ SwB cells were noted. Similarly, the frequency of these markers did not change between days 28-140 in SF2a- cells. The p values of these comparisons are summarized in **Supplementary Table 2**.

*Shigella* targets the intestinal mucosa, therefore, it is desirable for a vaccine to induce *Shigella*-specific immune cells able that migrate to gut associated lymphoid tissues (GALT). The adhesion molecule integrin α4β7 plays a crucial role in controlling lymphocyte migration to the intestine by binding to MAdCAM-1, which is expressed on Peyer’s patch high endothelial venules (HEV) and lamina propria venules, which are part of the GALT (49, 50). We thus assessed the expression of integrin α4β7 in SF2a+ and SF2a- SwB cells. Overall, SF2a+ SwB cells tended to express lower levels of integrin α4β7 than SF2a- SwB cells after vaccination. Statistically significant differences between SF2a+ and SF2a- cells were noted on days 28 and 140 (**Figure 4D**). We also assessed whether there were differences in IgG (**Figure 4E**) and IgA (**Supplementary Figure 2C**) expression between SF2a+ and SF2a- SwB cells. Overall, SF2a+ SwB cells tended to exhibit lower frequencies IgG expression than SF2a- SwB cells, especially on days 56, 84 and 140 (**Figure 4E**). SF2a+ and SF2a- SwB cells expressed IgA at similar frequencies (**Supplementary Figure 2C**).

To further explore the immunophenotypic characteristics of SF2a-specific B cells, we performed unsupervised analyses in concatenated SF2a+ SwB cells from all vaccinated volunteer timepoints, except day 0 since no SF2a+ SwB cells were identified at that timepoint (**Figure 2A**). Cells clusters rendered by a dimensionality reduction analysis (t-SNE) were assessed using X-shift. Three cell clusters were identified and the main distinctive feature between these clusters was the expression of IgG, IgA and IgG-IgA-) (Cluster 1, 2 and 3, respectively) (Cluster explorer) (**Figures 2B, C**). Interestingly, the IgG-IgA- cluster showed the largest frequency of SF2a+ SwB cells (**Figure 2B**; **Supplementary Figure 2B**).

### Differentiation into ASC and changes in expression of activation and homing markers

**Figure 5:**
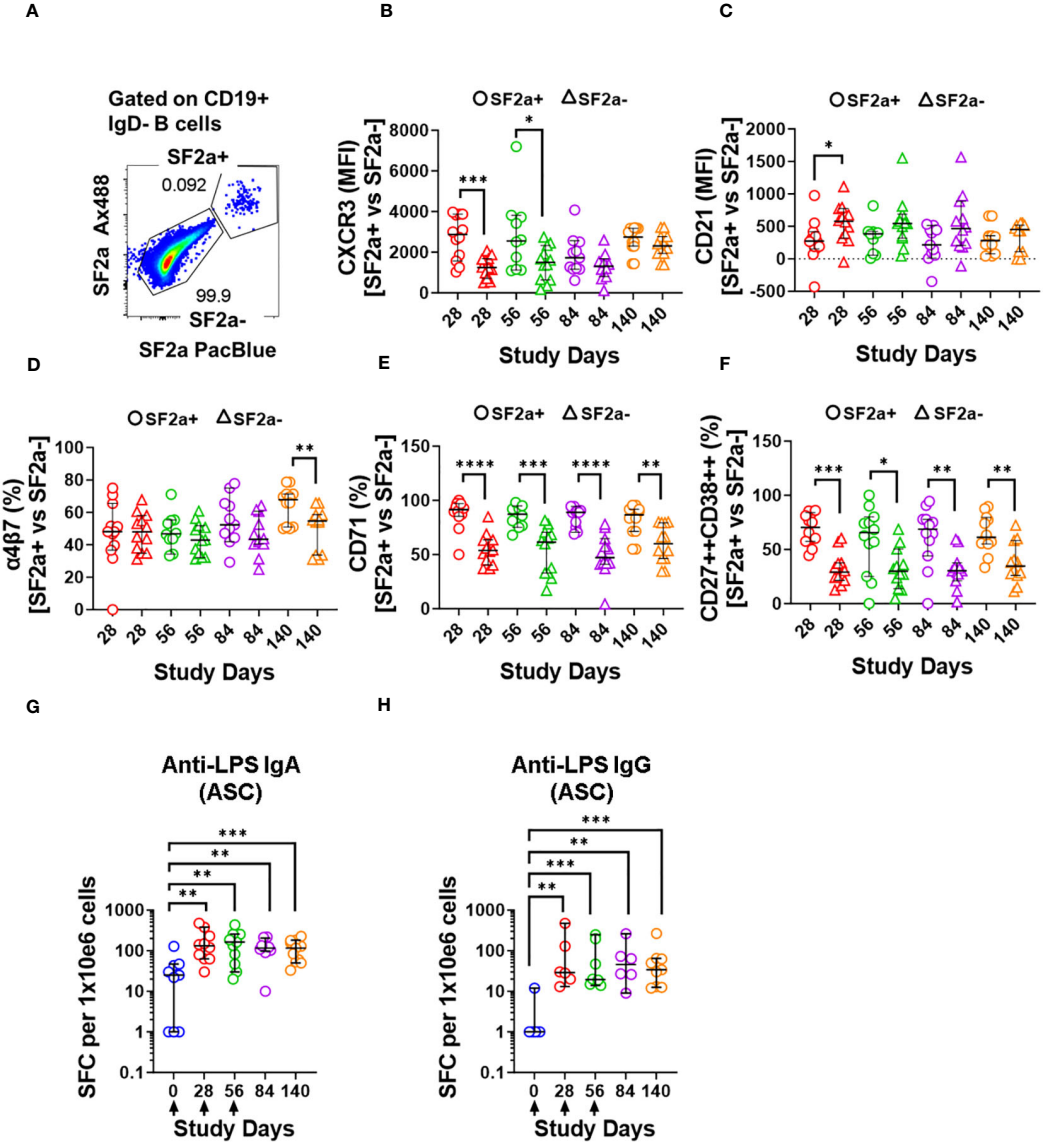
Characteristics of SF2a+ B cells in ASC-differentiated cells. Panel **(A)** shows an example of the gating used to identify SF2a+ and SF2a- in ASC-SwB cells. Data is from a representative volunteer post-vaccination. Panels **(B, C)** show the median fluorescence intensity (MFI) of CXCR3 and CD21 in SF2a+ and SF2a- ASC-SwB cells in SF2a-TT15 +/- Alum vaccinated volunteers. Panels **(D, E)** display the percentage of SF2a+ and SF2a- in ASC-SwB cells expressing integrin α4β7 and CD71. **(F)** displays the percentages of SF2a+ and SF2a- ASC-SwB cells with characteristics of plasmablast/plasma cells [CD27++ CD38++] (look also **Supplementary Figure 3B**). Panel **(G, H)** show the frequency of anti-LPS IgA and IgG spot forming cells -SFC- per 1x10e6 cells in volunteers that received SF2a-TT15 +/- Alum. Arrows in the X axis show the vaccination days. Panels **(B-H)** show the median and 95CI. The p values of panels **(B-F)** were calculated using unpaired t-tests (2-sided). *p<0.05, **p<0.005, ***p<0.001, ****p<0.0001. The p values of panels **(G, H)** were calculated using unpaired Mann Whitney tests (2-sided). **p<0.005, ***p<0.001.

One of the most critical aspects of vaccination is the induction of memory. Upon an antigen re-encounter, antigen-specific memory B cells are rapidly recruited to become ASC and these, in the case of *Shigella* vaccines, would be desirable to migrate to the gut. SF2a-specific SwB cells identified in PBMC showed an activated phenotype (high CXCR3 and low CD21) with limited ability to migrate to the gut, as compared to SF2a- SwB cells (**Figures 4B-D**). To explore whether the characteristics of these cells change when they actively secrete antibodies, we differentiated B cells from the vaccinees into ASC using a previously described protocol (ASC-transformed) (35–38) and assessed them by flow cytometry. The gating strategy was different from that used in PBMC and focused primarily in identifying SwB cells (ASC-SwB cells; CD19+/dim IgD-) (**Figure 3A**) since the differentiation process skews the populations towards plasmablasts and plasma cells phenotypes. Moreover, all these cells were expected to express high levels of activation and proliferation markers making it difficult to define ABCs or other B subsets. SF2a+ ASC-SwB cells were identified at every timepoint of the study, except day 0 (pre-vaccination) (**Figures 3B, C**). Day 28 appears to be the timepoint with the highest frequency of these cells. Interestingly, by day 140 the frequency of these cells was reduced, and no differences, as compared to day 0 were noted. Moreover, this timepoint (day 140) showed statistical differences compared to day 28 (**Figure 3C**; **Supplementary Table 3**). Since SF2a+ ASC-SwB cells were virtually absent at day 0, we report results of various activation/immunophenotyping markers from days 28 to 140. SF2a+ ASC-SwB cells (**Figure 5A**) showed significantly higher expression levels of CXCR3 than SF2a- ASC-SwB cells on days 28 and 56 (**Figure 5B**). SF2a+ ASC-SwB tended to express lower levels of CD21 than in SF2a- ASC-SwB cells across all timepoints, but statistical significance was only shown on day 28 (**Figure 5C**). SF2a+ ASC-SwB cells tended to have higher expression levels of integrin α4β7 than SF2a- ASC-SwB; however, statistical significance was shown only on cells from day 140 of the study (**Figure 5D**). Moreover, ASC SF2a+ SwB cells showed active proliferation as suggested by the higher expression levels of CD71 (**Figure 5E**) on days 28, 56, 84 and 140. We also assessed expression of markers associated with plasmablast and plasma cells differentiation (CD27++ and CD38++). Significantly higher percentages of CD38++CD27++ cells were observed in SF2a+ ASC-SwB cells than in SF2a- ASC-SwB cells (**Figure 5F**; **Supplementary Figure 3B**). We also compared changes in the expression of the integrin α4β7, CXCR3 and CD21 in SF2+ and SF2- SwB cells between PBMC and ASC-transformed cells. For these analyses, data from timepoints 28-140 were grouped (**Supplementary Figures 4A-C**). Integrin α4β7 expression increased when the cells were differentiated into ASC (**Supplementary Figure 4A**). Of note, SF2a+ ASC-SwB cells expressed higher levels of this molecule than SF2a- ASC-SwB cells, which contrasted with the expression of this marker in PBMC. Compared to PBMC, ASC-transformed expressed higher levels of CXCR3. Moreover, SF2a+ SwB cells in both PBMC and ASC expressed higher levels than SF2a- SwB cells (**Supplementary Figure 4B**). Finally, ASC differentiation decreased the expression of CD21 and SF2a+ SwB cells in both PBMC and ASC expressed the lowest levels of this marker, compared to SF2a- SwB cells (**Supplementary Figure 4C**).

Since we differentiated B cells into ASC, it was important to assess whether these cells were producing vaccine specific antibodies. To this end, we assessed the anti-LPS IgA and IgG production by ELISpot in the volunteers whose samples had sufficient cells to perform these assays. Anti-LPS IgA was assessed in 9-12 vaccinated volunteers per timepoint and in 3-4 placebo volunteers per timepoint. Anti-LPS IgG was assessed in 6-9 vaccinated and 1-3 placebo volunteer per timepoint (**Supplementary Figure 5**). In vaccinated volunteers, the frequency of anti-LPS IgA and IgG ASC increased significantly after day 0 (**Figures 5G, H**). None of the other timepoints showed statistical differences among them (**Supplementary Table 4**).

## Discussion

The B cell compartment is critical for protection against *Shigella* infections (17–19, 38, 51–55). To date, development of humoral responses after *Shigella* infection or vaccination has been assessed using classical immunological methods such as ELISA and ELISpot. However, a better understanding of the immunophenotypic features of *Shigella*-specific B cells is missing. The use of molecular probes labeled with fluorescent dyes or other tags has been employed to study, in detail, the development of humoral immunity to viruses such as influenza virus and SARS-CoV-2 (56–62), but not for bacteria, to date. To address this gap, and as a proof-of-principle study, we used fluorescently labeled SF2a bacteria to identify *Shigella*-specific B cells in specimens from human volunteers who had received the vaccine candidate SF2a-TT15 (21).

SF2a-TT15 is a synthetic carbohydrate conjugate vaccine, whereby the glycan component is a unique 15mer oligosaccharide designed to achieve SF2a O-antigen functional mimicry. We opted to use whole bacteria as “bait” to identify antigen-specific B cells, to ensure that this vaccine induced B cells able to recognize the LPS from wild-type SF2a. Our fluorescent labeling strategy targets free amines on surface proteins of the bacteria; thus, the O-antigen was not modified during the labeling process and maintained its natural structure. We incorporated bacteria labeled with two different fluorochromes (Ax488 and PB) into high-color flow cytometry panels to reduce the possibility of measuring non-specific B cell binders which have the potential to obscure the results (**Figure 1A**; **Supplementary Figures 1E, F**). Using this strategy, we identified SF2a-specific B cells in the B memory cell compartment at every timepoint (28-140 days) after the first immunization, except day 0 (pre-vaccination) (**Figures 1B, C**; **Supplementary Figure 2A**). The absence of SF2a+ B cells in the plasmablast and ABC compartments is likely due to the timepoints available for these assessments. Antigen-specific B cells are usually identified in the plasmablast and ABC compartments ~8 days and ~2 weeks after vaccination/infection, respectively. Samples corresponding to these timepoints were not available from these vaccinees.

In PBMC, SF2a+ SwB cells expressed high levels CXCR3 and low levels of CD21. Expression of CXCR3 has been associated with the ability of cells to migrate to inflamed tissues (43–46). On the other hand, CD21 downregulation, in the context of antigen-specific B cells, has been associated with cells that left the germinal center (GC) and are primed for plasma cell differentiation (48). Importantly, CD21-low antigen-specific B cells (63, 64) have also been shown to express high levels of T-bet (65) and FcLR5 (66). CXCR3 is a transcription target of T-bet and a recent paper showed that co-expression of CXCR3 with T-bet and FcLR5 distinguishes a subset of effector memory B cells able to rapidly differentiate into antibody secreting cells (66). Taken together, the cells we identified (SF2a+ SwB cells) in SF2a-TT15 vaccinees are likely effector memory B cells, which are primed to become ASC. This would be the first report of these cells being elicited by vaccination for an enteric bacterial pathogen.

IgG and IgA expressing B cells were identified within SF2+ SwB cells in PBMC (**Figure 1E**; **Supplementary Figure 2C**). Considering that SF2a-TT15 was delivered intramuscularly, it is not surprising that IgG was the predominant isotype. The unsupervised analysis suggested that IgG was one-third more abundant than IgA (~30% vs. 20% respectively) in SF2a+ SwB cells (**Figure 2B**, group 1 vs. group 2). A significant percentage of SF2a+ B cells were IgG-IgA- (**Figure 2**; **Supplementary Figure 2B**). The significance of this finding is unclear at this time. Of note, SF2a- SwB cells also showed a proportion of IgG-IgA- cells (**Supplementary Figure 3B**). Other studies have shown similar results (significant proportion of IgG-IgA- B cells) in non-antigen-specific B cells (67–69), but no clear explanation for these findings has been provided. Future studies will address this aspect in more detail.

One critical function of memory B cells is reactivation and differentiation into ASC once a re-infection occurs. We differentiated B cells from SF2a-TT15 vaccinees into ASC to determine their ability to secrete anti-LPS IgA and IgG by ELISpot and the immunophenotypic characteristics of SF2a+ ASC-SwB cells by flow cytometry. As expected, SF2a+ ASC-SwB cells were identified only in vaccinated volunteers (**Figures 3B, C**; **Supplementary Figure 3**). These cells continued to express high levels of CXCR3 (significant on days 28 and 56) and low levels of CD21 (significant on day 28), suggesting that they maintained the characteristics of effector memory cells. Further evidence of this came from comparisons of the degree of expression of these markers between PBMC and ASC-transformed [cumulative analysis including all datapoints after vaccination (days 28 to 140)]. ASC differentiation increased the expression of CXCR3 and downregulated CD21; however, SF2a+ ASC-SwB cells showed the highest and lowest levels of CXCR3 (**Supplementary Figure 4B**) and CD21 expression (**Supplementary Figure 4C**), respectively. Interestingly, the frequency of SF2a+ ASC-SwB cells appeared to be reduced in the later timepoints, which might suggest that in some volunteers the memory compartment was contracting. Nevertheless, in several volunteers, memory B cells with the ability to produce anti-LPS IgG and IgA antibodies were still present at this timepoint, as shown by the ASC assessments (**Figures 5G, H**).

These results are concordant with the memory responses reported by Cohen et al. (21) in the Phase I trial, where memory responses, assessed by ELISpot, were shown to be present until day 140. A long-term follow-up of these volunteers was recently published showing that memory B cells (ELISpot) returned to almost baseline levels two years after vaccination. Nevertheless, up to 89% of the volunteers that received 10 μg OS maintained >4-fold titers of anti-SF2a LPS IgG compared to their pre-vaccination levels (70). It is important to note that antibodies detected in sera long after vaccination are produced by long-lived plasma cells (LLPC) that reside in the bone marrow (71, 72). LLPC are not memory B cells since the latter are defined as antigen-experienced quiescent B cells that can become re-activated to rapidly produce antibodies (73–75). Hence, assessment of circulating memory B cells provide only partial information on the B memory compartment. Moreover, the VDJ sequences in circulating memory B cells and serum antibodies do not always fully match due to their different cell origins. Similarly, the frequencies of circulating B cells do not always correlate with the serum antibody titers for a given antigen. We believe that the SF2a+ SwB cells identified in this manuscript represent a group of memory cells ready to be differentiated into antibody secreting cells in recall responses.

Another notable change of SF2a+ ASC-SwB cells, compared to PBMC, involved the upregulation of integrin α4β7 (**Supplementary Figure 4A**; **Figure 4D** vs. **5D**). The cumulative datapoint analysis of PBMC vs. ASC showed that SF2a+ ASC-SwB cells expressed higher levels of integrin α4β7 than SF2a- ASC-SwB (**Supplementary Figure 4A**). This suggests that vaccination with SF2a-TT15 induces B cells that express integrin α4β7, but these are probably in circulation only for few days after vaccination. Likely, we did not identify them in circulation (PBMC samples) because by the time of blood sampling, these cells have already migrated to GALT. Nevertheless, re-activation of SF2a+ SwB cells (ASC differentiation) triggered the expression of this marker again, confirming the potential of these cells to migrate to the GALT once they start to secrete antibodies. It is also possible that SF2a-TT15 induces only limited expression of integrin α4β7 and that this marker is upregulated in ASC-transformed cells due to the intense *in vitro* stimulation. Future studies in which plasmablasts from SF2a-TT15 recipients are sorted based on the expression of i) gut-homing (e.g., integrin α4β7) or ii) peripheral lymphoid organ-homing (e.g., CD62L) markers and assessed for anti-LPS antibody production (76) could provide more clear answers.

As expected, all polyclonally stimulated B cells expressed high levels of CD71. However, in SF2a+ ASC-SwB cells the expression of this marker was double than in SF2a- ASC-SwB cells (**Figure 3E**), which suggests that SF2a+ cells were proliferating at higher rates than other differentiated non-specific ASC. Moreover, in the ELISpot assay, it was clear that anti-LPS IgA and IgG ASC increased in frequency after day 0 (**Figures 5G, H**).

Overall, we developed a method to identify circulating SF2a-specific B cells. Using this approach, we demonstrated that SF2a-TT15 induced circulating SF2a+ SwB cells, reflecting the induction of memory cells. These results confirm and markedly expand previous published data by Cohen et al. (21) that showed the induction of memory B cells (by ELISpot) in these volunteers. Herein, the proof-of-concept for the potential of this novel technology was achieved in samples from volunteers enrolled in a first-in-human study for SF2a-TT15, a synthetic carbohydrate-based vaccine candidate now being assayed in phase 2 clinical trials. This methodology is less labor intensive than classic ELISpot methods and could be used to easily track the presence of antigen-specific memory B cells in circulation after vaccination or infection with SF2a. Moreover, it can provide a wealth of information on the characteristics of antigen-specific B cells to enteric bacteria and allow detailed examination of the dynamic development of SF2a-specific B cells involving other subsets (e.g., ABCs). For these studies, appropriate timepoints will be collected in the future. Moreover, this methodology will be combined with other antigen probes already developed in our lab (e.g., LPS fluorescent nanoparticles (31, 32), Core-O-polysaccharides-FITC, IpaB-PacBlue, IpaD-AlexaFluor700) to study the development of B cell immunity to multiple specific *Shigella* antigens in specimens of volunteers vaccinated and then challenged with wild-type *Shigella*. The probe used to identify antigen-specific B cells (whole bacteria, LPS, or purified protein) will depend on the type of vaccine used and the antigen of interest. These reagents will also be combined with next generation sequencing for single-cell assessments of clonality to diverse antigens, and the generation of recombinant antibodies which can be used as standards across multi-center studies.

## Data availability statement

The raw data supporting the conclusions of this article will be made available by the authors, without undue reservation.

## Ethics statement

The requirement of ethical approval was waived by The University of Maryland Baltimore IRB for the studies on humans because the specimens received were de-identified; hence, this project was determined to be “Not human research”. The studies were conducted in accordance with the local legislation and institutional requirements. Written informed consent for participation was not required from the participants or the participants’ legal guardians/next of kin in accordance with the national legislation and institutional requirements. The human samples used in this study were acquired from primarily isolated specimens collected as part of a previous study for which ethical approval was obtained.

## Author contributions

FT: Conceptualization, Formal Analysis, Funding acquisition, Investigation, Methodology, Supervision, Writing – original draft, Writing – review & editing, Project administration, Resources. JH: Formal Analysis, Methodology, Writing – original draft, Writing – review & editing. SM: Resources, Writing – review & editing. LM: Data curation, Writing – review & editing. AP: Data curation, Writing – review & editing. DC: Resources, Writing – review & editing. MS: Conceptualization, Funding acquisition, Investigation, Resources, Supervision, Writing – original draft, Writing – review & editing.
