# Supplementary material for: Further characterization of Shigella-specific (memory) B cells induced in healthy volunteer recipients of SF2a-TT15, a Shigella flexneri 2a synthetic glycan-based vaccine candidate
Source: Front Immunol. 2023 Oct 31;14:1291664. doi: 10.3389/fimmu.2023.1291664 (PMC10653583; doi:10.3389/fimmu.2023.1291664)
Supplement: Supplementary file 1 [file DataSheet_1.docx]

**Supplementary Figures**:

**
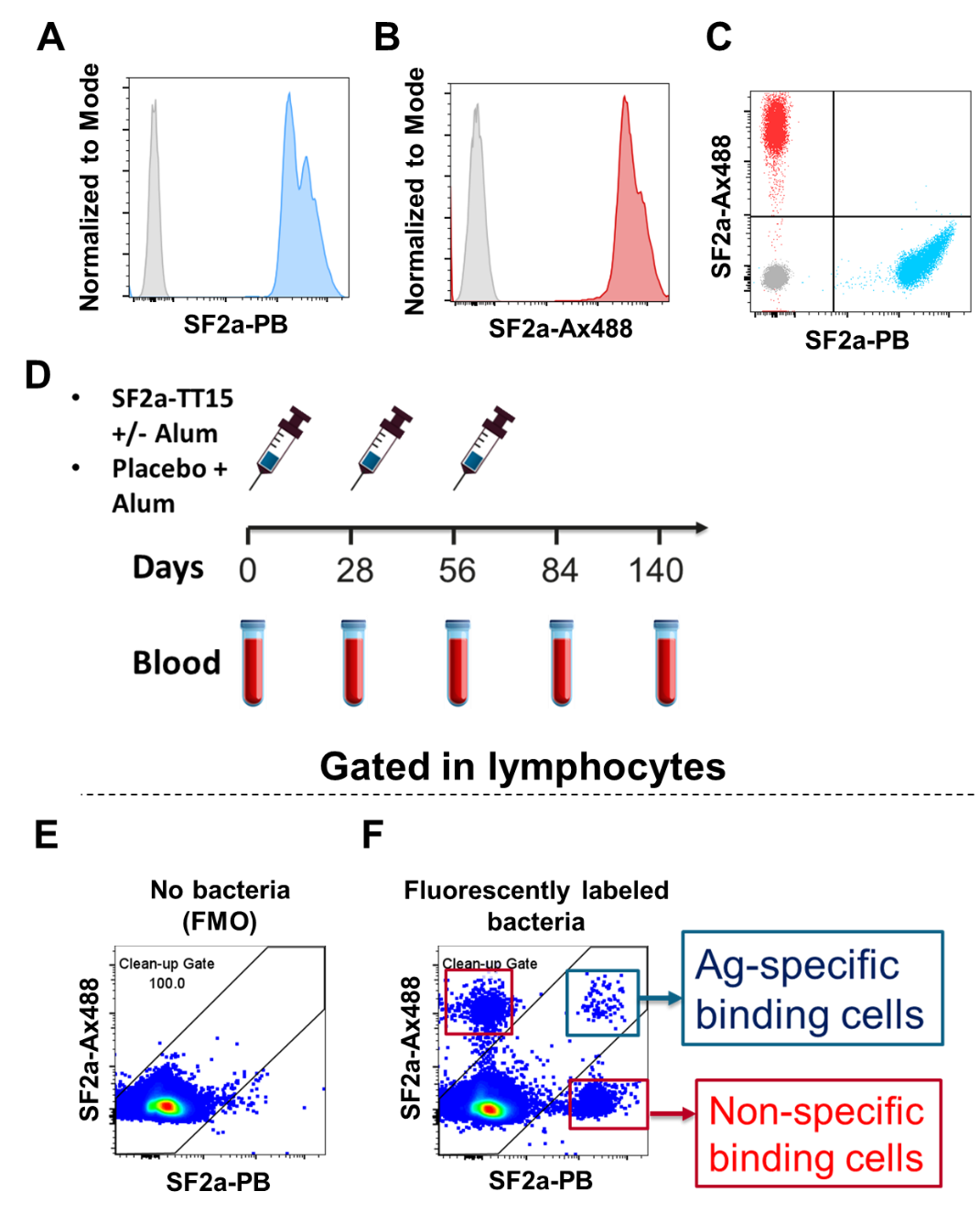
**

**Supplementary Figure 1. Fluorescently labeled bacteria, study outline and non-specific clean-up gates.** Panel **A** shows an overlay of unlabeled (gray histogram) and Pacific Blue (PB; blue histogram) labeled *Shigella flexneri* 2a [2457T] (SF2a). Panel **B** shows an overlay of unlabeled (gray histogram) and Alexa Fluor 488 (Ax488; red histogram) labeled SF2a. Panel **C** shows a dot plot overlay of SF2a-PB (Blue), SF2a-Ax488 (red), and unlabeled SF2a (gray). Virtually all bacteria were labeled with the fluorescent dyes. Panel **D** shows a schematic of vaccination days and days in which blood samples (PBMC) were collected from the volunteers. Panel **E** shows a sample in which labeled SF2a were not added (FMO) and panel **F** shows identification SF2a-binding B cells in an immunized volunteer. Note that SF2a-specific cells are considered those that bind to both PB- and Ax488-labeled SF2a (dual binders; Ag-specific binding cells). Cells that bind only to PB- or only to Ax488-labeled bacteria are considered non-specific binders and were eliminated in the gating strategy by introducing a clean-up gate. FMO: Fluorescence minus one.

**
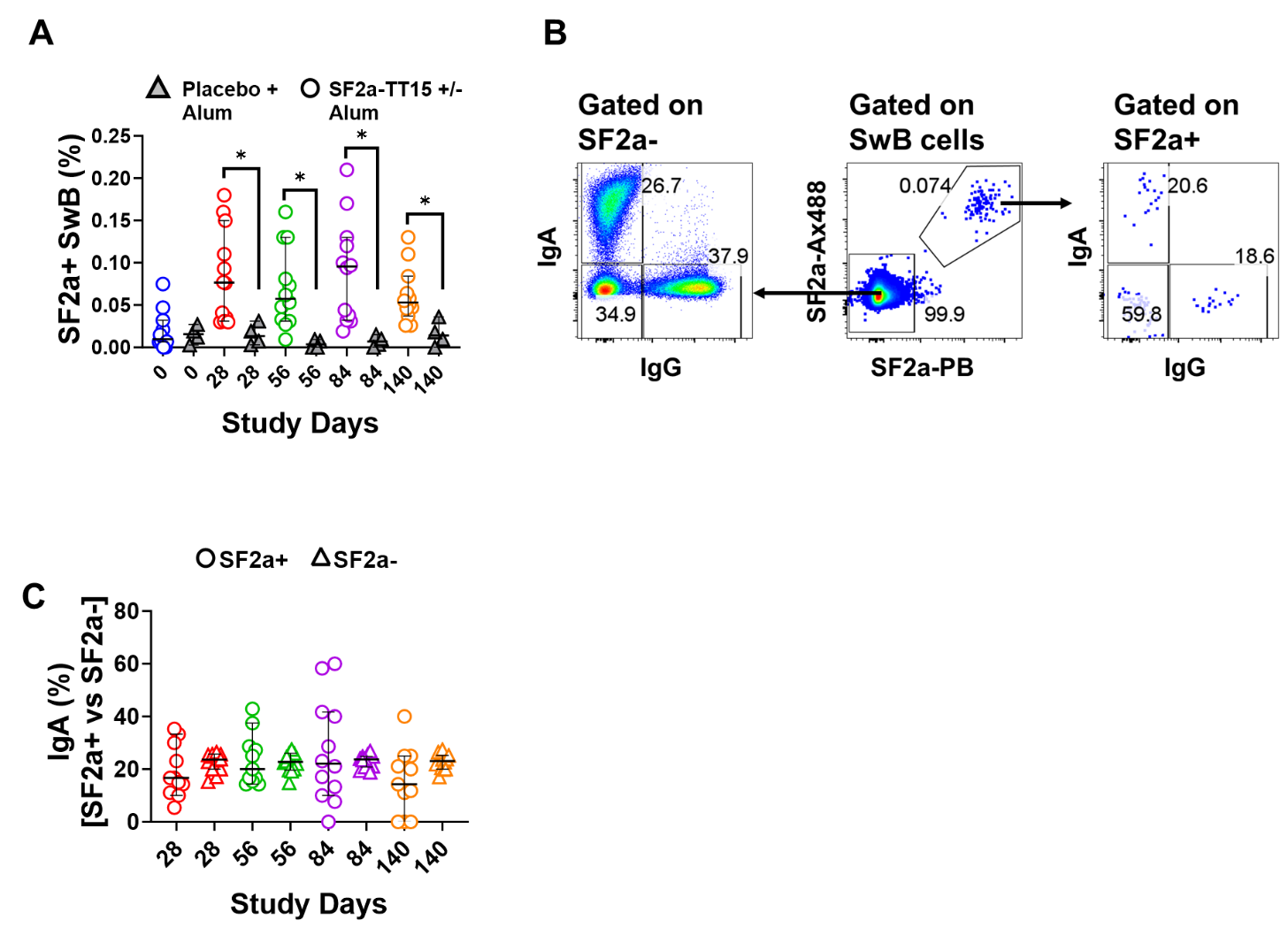
**

**Supplementary Figure 2. Additional data from SF2a+ SwB cells in PBMC.** Panel **A** shows that SF2a+ cells within SwB cells were virtually absent before the start of the study (day 0). These cells remained absent at all timepoints in volunteers that received Placebo + Alum. In SF2a-TT15 +/- alum vaccinated individuals, the frequency of SF2a+ SwB cells increased after day 0. The median and 95CI are shown. The p values were calculated using unpaired t-tests (2-sided). *p<0.05. Panel **B** shows the gating strategy used to determine the expression of IgG and IgA in SF2a+ and SF2a- cells within SwB cells. Panel **C** shows that the expression of IgA in SF2a+ and SF2a- cells within SwB cells (days 28-140) was similar.

**
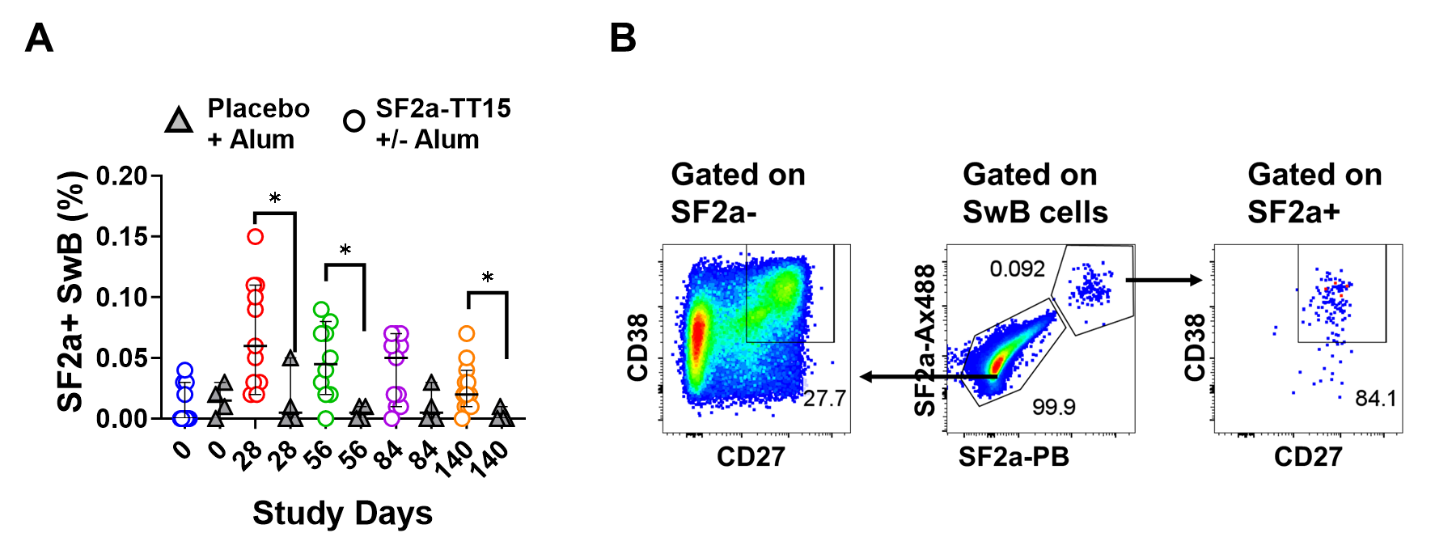
**

**Supplementary Figure 3. Additional data from SF2a+ ASC-SwB cells.** Panel **A** shows that SF2a+ cells within ASC-SwB cells were virtually absent before the start of the study (day 0). These cells remained absent at all timepoints in volunteers that received Placebo + Alum. In SF2a-TT15 +/- alum vaccinated individuals, the frequency of SF2a+ SwB cells increased in most individuals after day 0. The median and 95CI are shown. The p values were calculated using unpaired t-tests (2-sided). *p<0.05. Panel **B** shows the gating strategy used to identify cells expressing high levels of CD27 and CD38 (CD27++ CD38++) in SF2a+ and SF2a- SwB cells. CD27++ CD38++ are phenotypic characteristics of plasmablasts and plasma cells.

**
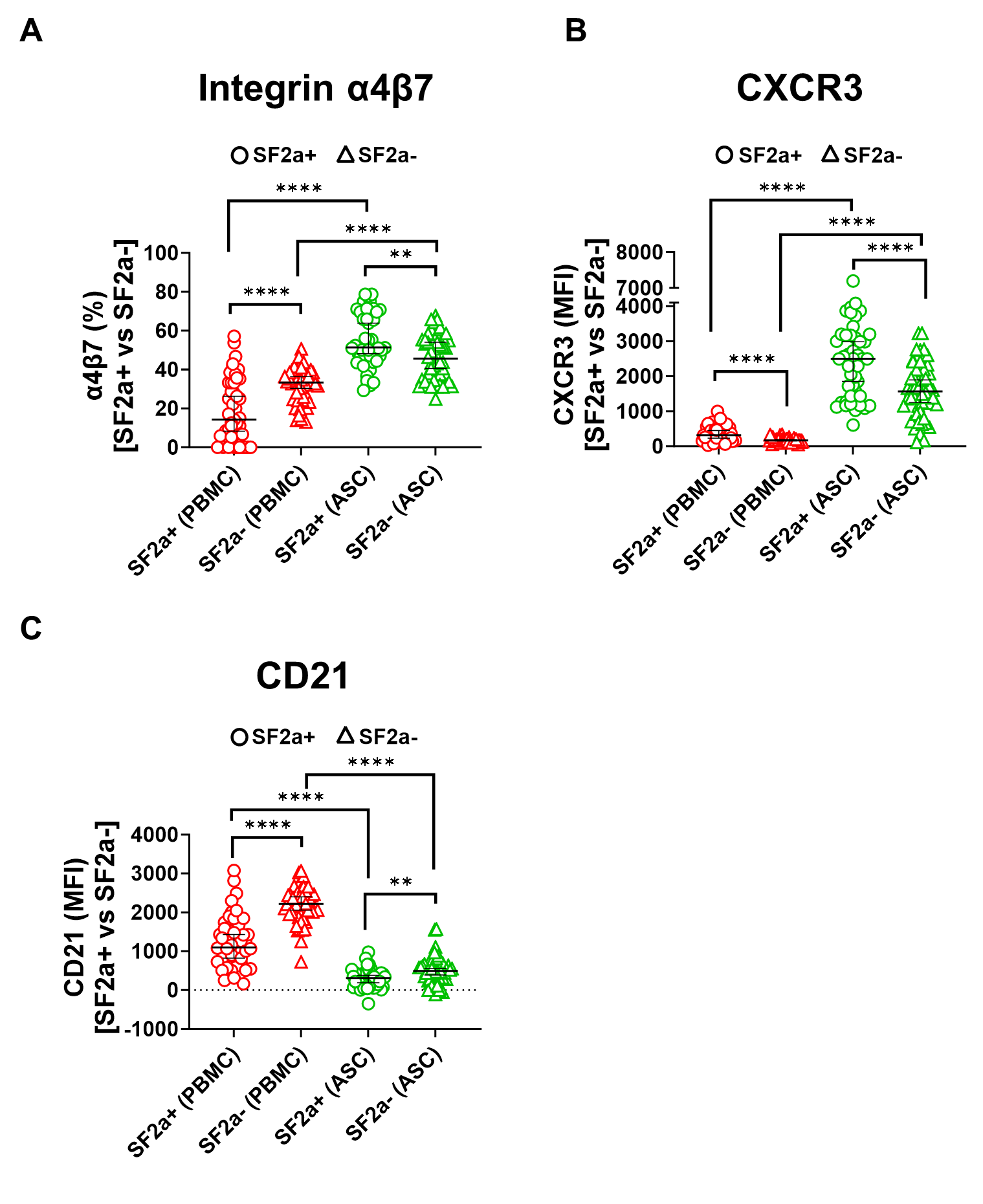
**

**Supplementary Figure 4. Expression of integrin α4β7, CXCR3, and CD21 in SwB cells from PBMC and ASC-differentiated cells.** Panels **A-C** show the expression of integrin α4β7, CXCR3, and CD21 in SF2a+ and SF2a- within the SwB cell compartment in PBMC and ASC-differentiated cells. The comparisons include all datapoints after vaccination (days 28 to 140). The graphs show the median and 95CI. The p values were calculated using unpaired t-tests (2-sided). *p<0.05, ** p<0.005, *** p<0.001, **** p<0.0001.

**
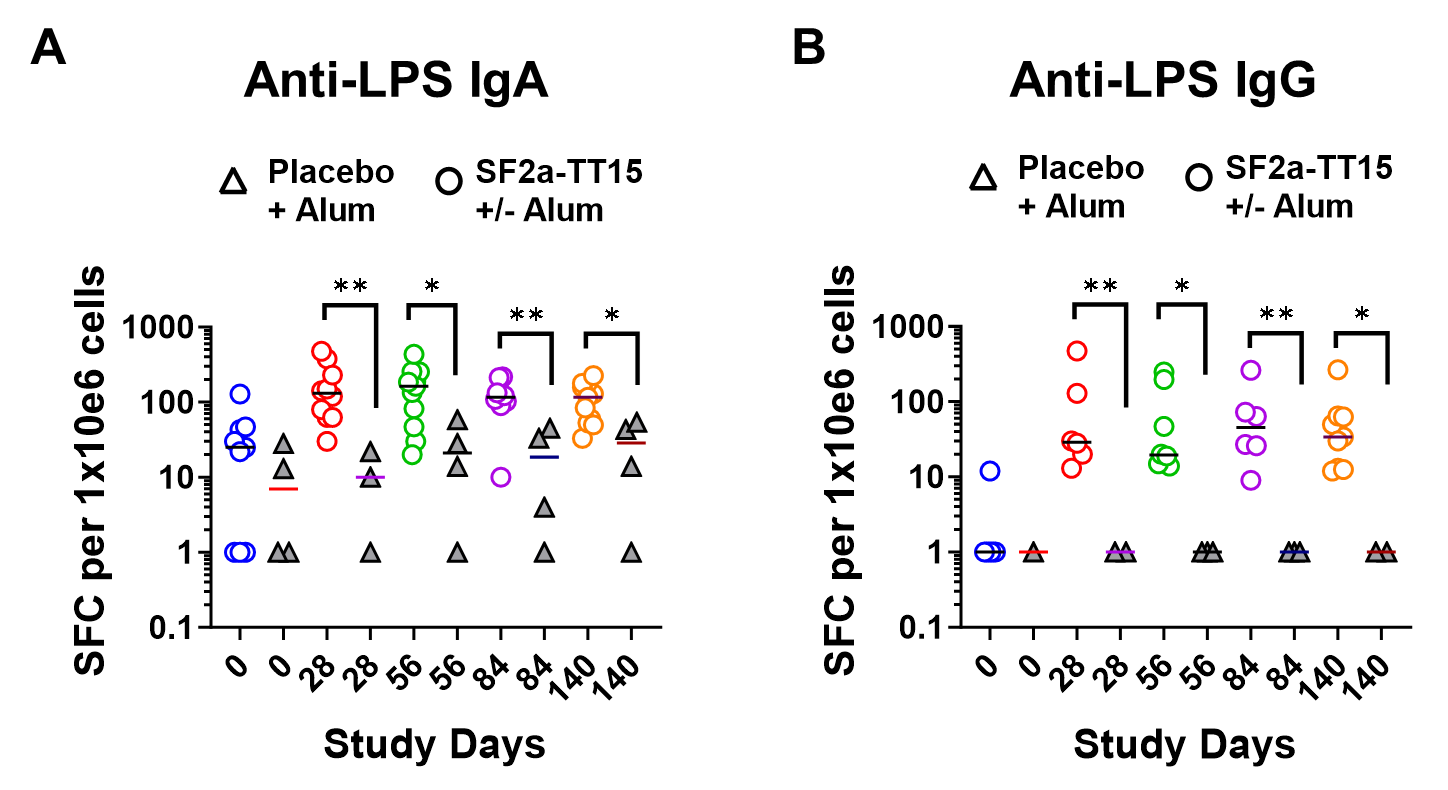
**

**Supplementary Figure 5. Anti-LPS IgA and IgG ASC.** Panels **A** and **B** show the frequency of cells producing anti-LPS IgA and IgG in SF2a-TT15 +/- Alum (circles) and Placebo + Alum (gray triangles) recipients. Note that the assay was performed only in volunteers with enough cells to perform these assays. The graphs display individual data and the mean (horizontal bar). The p values were calculated using unpaired Mann Whitney tests (2-sided). * p<0.05, ** p<0.005.

**Supplementary Table 1.** Statistical analysis of the frequency of SF2a+ SwB cells in diverse timepoints of the study. *P* values < 0.05 are considered significant.

|  | ***p* from Student's t-tests, unpaired, 2-sided** | | | | | |
| --- | --- | --- | --- | --- | --- | --- |
|  | **Day 28 vs.**  **Day 56** | **Day 28 vs.**  **Day 84** | **Day 28**  **vs.**  **Day 140** | **Day 56 vs.**  **Day 84** | **Day 56 vs.**  **Day 140** | **Day 84**  **vs.**  **Day 140** |
| **SF2a+ SwB** | 0.4914 | 0.8014 | 0.2181 | 0.3618 | 0.6074 | 0.1527 |

**Supplementary Table 2**. Statistical analysis of CD21 and CXCR3 expression in SF2a+ and SF2a- SwB cells in diverse timepoints of the study. *P* values < 0.05 are considered significant.

|  | ***p* from Student's t-tests, unpaired, 2-sided** | | | | | |
| --- | --- | --- | --- | --- | --- | --- |
|  | **Day 28 vs.**  **Day 56** | **Day 28 vs.**  **Day 84** | **Day 28**  **vs.**  **Day 140** | **Day 56 vs.**  **Day 84** | **Day 56 vs.**  **Day 140** | **Day 84**  **vs.**  **Day 140** |
| **CXCR3 in SF2a+ SwB cells** | 0.9418 | 0.6816 | 0.2071 | 0.792 | 0.2447 | 0.0838 |
| **CXCR3 in SF2a- SwB cells** | 0.6684 | 0.5296 | 0.2675 | 0.9106 | 0.5286 | 0.5214 |
| **CD21 in SF2a+ SwB cells** | 0.0849 | 0.1862 | 0.0576 | 0.3492 | 0.8871 | 0.2578 |
| **CX21 in SF2a- SwB cells** | 0.9657 | 0.8817 | 0.8817 | 0.878 | 0.6371 | 0.4517 |

**Supplementary Table 3.** Statistical analysis of the frequency of SF2a+ ASC-SwB cells in diverse timepoints of the study. *P* values < 0.05 are considered significant.

|  | ***p* from Student's t-tests, unpaired, 2-sided** | | | | | |
| --- | --- | --- | --- | --- | --- | --- |
|  | **Day 28 vs.**  **Day 56** | **Day 28 vs.**  **Day 84** | **Day 28**  **vs.**  **Day 140** | **Day 56 vs.**  **Day 84** | **Day 56 vs.**  **Day 140** | **Day 84**  **vs.**  **Day 140** |
| **SF2a+ ASC-SwB** | 0.1833 | 0.0718 | ***0.005*** | 0.5851 | 0.0601 | 0.1719 |

**Supplementary Table 4.** Statistical analysis of anti-LPS IgA and IgG ASC in diverse timepoints of the study. *P* values < 0.05 are considered significant.

|  | ***p* from Mann-Whitney, unpaired, 2-sided** | | | | | |
| --- | --- | --- | --- | --- | --- | --- |
|  | **Day 28 vs.**  **Day 56** | **Day 28 vs.**  **Day 84** | **Day 28**  **vs.**  **Day 140** | **Day 56 vs.**  **Day 84** | **Day 56 vs.**  **Day 140** | **Day 84**  **vs.**  **Day 140** |
| **Anti-LPS IgA (ASC)** | 0.9306 | 0.8085 | 0.4997 | 0.5555 | 0.4385 | 0.6396 |
| **Anti-LPS IgG (ASC)** | 0.5941 | 0.9372 | 0.842 | 0.4908 | >0.9999 | 0.8639 |
